# Complete genome sequence of potential plant growth-promoting *Bacillus altitudinis* strain AIMST-CREST03 isolated from paddy field bulk soil

**DOI:** 10.1128/mra.00261-24

**Published:** 2024-05-20

**Authors:** Sumitra Sivaprakasam, Nur Arisa Mohd Azim Khan, Tan Yee Fan, Yukgehnaish Kumarasan, Thomas Sicheritz-Pontén, Bent Petersen, Erneeza Mohd Hata, Ganesan Vadamalai, Sivachandran Parimannan, Heera Rajandas

**Affiliations:** 1 Center of Excellence for Omics-Driven Computational Biodiscovery (COMBio), AIMST University, Kedah, Malaysia; 2 Department of Plant Protection, Faculty of Agriculture, Universiti Putra Malaysia, Serdang, Selangor, Malaysia; 3 Center for Evolutionary Hologenomics, Globe Institute, University of Copenhagen, Copenhagen, Denmark; 4 Laboratory of Sustainable Agronomy and Crop Protection, Institute of Plantation Studies, Universiti Putra Malaysia, Serdang, Selangor, Malaysia; DOE Joint Genome Institute, Berkeley, California, USA

**Keywords:** genome, plant growth-promoter, *Bacillus*

## Abstract

We present the complete genome of a potential plant growth-promoting bacteria *Bacillus altitudinis AIMST-CREST03* isolated from a high-yielding paddy plot. The genome is 3,669,202 bp in size with a GC content of 41%. Annotation predicted 3,327 coding sequences, including several genes required for plant growth promotion.

## ANNOUNCEMENT

*Bacillus* is one of the most extensively studied plant growth-promoting (PGP) bacteria contributing significantly to the development of bio-formulations for field applications particularly in crops like paddy plants (1, 2). Here, we report the complete genome of a potential PGP *Bacillus altitudinis* strain AIMST-CREST03 (3–6) isolated from a paddy bulk soil sample from a high-yielding paddy plot at Kampung Gajah, Perak, Malaysia (4.1841° N, 100.9389° E) on 1 July 2022. Thirty gram of submerged bulk soil was collected at a depth of 0–20 cm.

The strain was initially isolated on a nutrient agar plate after subjecting 2.5 g of bulk soil to serial dilution in sterile water, ranging from 10^−1^ to 10^−8^ (7, 8). To achieve pure colonies, the strain required re-streaking only once. A single colony was then cultivated in 5 mL of fresh Luria-Bertani Broth at 37°C overnight prior to genomic DNA extraction using the GeneJET Genomic DNA Purification Kit (ThermoFisher) following the manufacturer’s instructions.

The DNA library (non-fragmented and non-size-selected) was prepared with the SQK-NBD114.24 native barcoding kit (Oxford Nanopore Technologies, UK) following the manufacturer’s instructions. The sample was subjected to native barcode ligation followed by adapter ligation using the NEBNext Quick Ligation Module (NEB, USA) as per the manufacturer’s instruction. The prepared library was then sequenced with Oxford Nanopore Technology’s MinION platform, with R10.4.1 flow cell. A recurrent neural network-based basecaller, Guppy v6.5.7 (high-accuracy model), was used (9). A total of 229,124 raw reads with an N50 value of 7,699 were generated. The raw reads were trimmed with Porechop v0.2.4 (10) and subjected to *de novo* assembly with Flye v2.9.2 (11). The resulting contig was polished with Medaka v1.8.1 (https://github.com/nanoporetech/medaka). The publicly available linked resource (CP145438) was annotated with PGAP v6.6 (12). Default parameters were used for all the analyses unless otherwise stated.

In line with Flye’s output, the bacterial genome assembly visualization on Bandage v0.9.0 (13) also indicated that it was a circular chromosome. A single contig of 3,669,202 bp with GC content of 41% and mean coverage of 400× was achieved. PGAP predicted that the genome encodes 3,824 genes including 3,327 coding sequences, 23 rRNA genes (5S, 16S, and 23S), 81 tRNAs, 5 non-coding RNAs, and 388 pseudogenes. A relatively high number of pseudogenes was predicted due to frameshifts (354 of 388), suggesting that some basecalling errors may remain in the final genome, and this is a known limitation of Oxford Nanopore Technology (ONT)-only assemblies (14). A blast search against the NCBI Nucleotide database identified *B. altitudinis* NC3 DNA as the top hit and average nucleotide identity analysis on EzBioCLoud (updated on 23 August 2023) (15) indicated 98.59% sequence similarity between the genomes.

PGAP annotation predicted several genes necessary for auxin biosynthesis including Indole-3-glycerol phosphate, anthranilate phosphoribosyltransferase, phosphoribosylanthranilate isomerase, and tryptophan synthase (16, 17). Genes coding for siderophore biosynthesis and transport were also identified. Siderophore production can promote plant growth by enhancing iron availability and providing phytosanitary protection (18). Aside from that, genes coding for biosynthesis of volatile organic compounds (butanediol and acetoin) known to promote growth by regulating ethylene, and cytokinin homeostasis were also predicted (19).

## Data Availability

The complete genome sequence of *Bacillus altitudinis* strain AIMST-CREST03 has been deposited in NCBI GenBank under the BioProject ID PRJNA1077217, BioSample ID SAMN39960593, SRA ID SRR28002519, and GenBank accession CP145438.
